# An Evaluation of Reference Bite Force Values: Investigating the Relationship Between Dental Prosthetic Restoration and Bite Force in a Cross-Sectional Study

**DOI:** 10.3390/jcm14082723

**Published:** 2025-04-15

**Authors:** Ina Nitschke, Celine Moede, Andreas Koenig, Bernhard A. J. Sobotta, Werner Hopfenmüller, Julia Jockusch

**Affiliations:** 1 Gerodontology Section, Department of Prosthetic Dentistry and Materials Science, Leipzig University, Liebigstraße 12, 04103 Leipzig, Germany; ina.nitschke@medizin.uni-leipzig.de (I.N.); celine.moede@medizin.uni-leipzig.de (C.M.); bernhard.sobotta@medizin.uni-leipzig.de (B.A.J.S.); 2 Materials Science Section, Department of Prosthetic Dentistry and Materials Science, Leipzig University, Liebigstraße 12, 04103 Leipzig, Germany; 3 Institute of Biometry and Clinical Epidemiology, Charité-Universitätsmedizin Berlin, Corporate Member of Freie Universität Berlin, Humboldt-Universität zu Berlin, Berlin Institute of Health, 10117 Berlin, Germany; werner.hopfenmueller@charite.de; 4 Department of Prosthetic Dentistry and Senior Dentistry, Brandenburg Medical School (Theodor Fontane), 14776 Brandenburg an der Havel, Germany

**Keywords:** bite force, masticatory performance, prosthetic restorations, occlusal force, dental status, prosthetic treatment outcome

## Abstract

**Objectives:** This study aimed to establish reference values for bite force in individuals with various prosthetic restorations and to examine the relationship between prosthetic treatment groups (PTGs) and bite force as an indicator of masticatory muscle function. **Materials and Methods:** In a cross-sectional study from November 2021 to March 2023, 198 participants aged 18 to 95 years were recruited from multiple dental and geriatric centers. The participants were assigned to seven PTGs based on their dental and prosthetic statuses. Bite force was measured using the Occlusal Force Meter GM10, with three recordings on each side of the jaw, and analyzed using ANOVA. **Results:** The bite force decreased with fewer teeth and the transition from fixed to removable dentures. Fully dentate participants exhibited the highest bite forces, differing significantly from the other groups (*p* < 0.001). For the fully dentate individuals (547 ± 240 N), the bite force decreased progressively with the extent of prosthetic restoration, reaching 55 ± 45 N in edentulous individuals with complete dentures in both jaws. However, edentulous participants with two interforaminal implants demonstrated higher bite forces than those with partial dentures. **Conclusions:** Bite force is significantly impacted by prosthetic restoration type. Fully dentate individuals have the highest bite forces, while edentulous patients with implant-supported dentures also show considerable bite forces, similar to those with partial dentures.

## 1. Introduction

Oral health and the ability to chew freely can not only determine good quality of life, but also general health and the prevention of malnutrition-related problems and diseases [1,2,3,4,5]. Tooth loss and poor oral health affect food choices and nutrient intake and increase the risk of systemic disease and malnutrition, potentially leading to frailty and sarcopenia [6,7,8,9].

The chewing process is influenced by several factors, including oral health, dental status, number of support zones—whether they are formed by natural teeth or dentures—type and quality of dentures, masticatory muscles, and bite force [10,11]. The number of antagonistic tooth contacts and the bite force appear to have the greatest influence on chewing [11,12,13,14].

Chewability can be assessed by chewing tests or questionnaires. While chewing tests provide a degree of objectivity in evaluating masticatory performance, questionnaires help to determine the subjective chewing ability. The maximum bite force (MBF) can be used as a parameter to indicate the functional status of the masticatory system. Factors reported to affect bite force include gender, age, height, weight, and general health [15,16,17,18]. However, occlusal contact is the factor with the greatest influence on bite force [19]. The higher the number of teeth and contacts made by the teeth, the higher the bite force [20].

Both bite force and chewing efficiency—which both contribute to chewing function—play crucial roles in oral function. A higher bite force and chewing efficiency contributes to effective food breakdown, facilitating bolus formation and enhancing digestion. Conversely, a reduced bite force and compromised chewing efficiency may lead to inadequate food comminution, resulting in an increased gastrointestinal burden and impaired nutrient absorption. Additionally, individuals with a lower bite force or chewing efficiency often adapt their dietary choices by favoring softer foods, which can, over time, lead to an imbalanced diet and potential nutritional deficiencies.

One of the key purposes of dental treatment is to improve oral function by restoring dental status with fillings and fixed or removable dentures [21]. It is also possible to improve chewing function, i.e., bite force and chewing efficiency, through physiotherapeutic training of the masticatory muscles [22]. Nevertheless, denture wearers show reduced muscle activity, muscle strength, and chewing ability [20,23,24,25].

The objectives of the study were, firstly, to determine the reference values of bite force for participants with different prosthetic restorations (study part A), and secondly, to demonstrate the relationship between the type of prosthetic restoration and bite force as an objectively measurable parameter and indicator of the functional state of the masticatory muscles (study part B). It could be used to compare the individually measured bite forces for a PTG with the patient’s bite force and to show the patient simply that the bite force with his or her restoration is below the norm. A bite force below the normative range may indicate impaired oral function, potentially leading to difficulties in food comminution, altered dietary habits, and a decline in overall chewing efficiency. This, in turn, could impact digestion and nutrition, highlighting the clinical relevance of assessing bite force in prosthetic rehabilitation.

It was hypothesized that the bite force would decrease with the change in the type of prosthetic restoration, characterized by a reduction in the number of teeth or antagonistic contacts and a change in the prosthetic restoration from fixed to removable and vice versa. The null hypothesis (H_0_) states that there is no significant difference in bite force between different prosthetic treatment groups, whereas the alternative hypothesis (H_1_) posits that bite force varies significantly depending on the type of prosthetic restoration and dental status.

## 2. Materials and Methods

### 2.1. Study Population and Sampling Strategy

A cross-sectional study was performed in the period from November 2021 to March 2023. The study included participants aged 18 and over, regardless of their dental and prosthetic status. An upper age limit for participation in the study was not specified.

To minimize selection bias, the participants were recruited consecutively and voluntarily from different clinical settings: participants were recruited from the patient clientele of the Department of Prosthetic Dentistry and Materials Science, Leipzig University, a private dental office, and a geriatric rehabilitation clinic in Berlin to be able to include a wide scope of prosthetic dental restorations across different age groups and care contexts.

The sample size was based on feasibility and the goal of covering a broad range of prosthetic treatment groups (PTGs). A formal sample size calculation was not performed due to the exploratory nature of the study. However, efforts were made to include at least 20 participants per PTG.

Excluded from participation were participants with known or diagnosed temporomandibular disorders, including parafunctional habits detected during the study examination. In addition, participants were excluded if any acute treatment needs were observed, such as pain or abscesses, facial paralysis, or neuralgia. Participants with congenital mental retardation or acquired cognitive impairment were also excluded from the study, as it was necessary for patients to be able to exactly follow the instructions for carrying out the masticatory force measurement.

A clinical examination of the dental status was carried out using the DMFT index (D—decayed, M—missing, F—filled, T—teeth) [26] and an assessment of the occlusal support according to the Eichner index [27], as well as the number of support zones and the evaluation of the denture quality according to Marxkors [28].

### 2.2. Efforts to Address Potential Sources of Bias

To minimize potential sources of bias, several measures were implemented. All bite force measurements were performed by a single calibrated examiner to ensure consistency. The blinding of the examiner was not feasible due to the nature of the study design, but participants were not informed about the normative bite force values in order to avoid performance bias during measurement. To reduce selection bias, participants were recruited from three different care settings (university clinic, private practice, geriatric rehabilitation facility), ensuring a wide spectrum of prosthetic restorations and age groups. Patients with known parafunctional habits such as bruxism or clenching were excluded, either based on self-report or clinical signs, to avoid distortion of the bite force values.

### 2.3. Measurements

**Figure 1:**
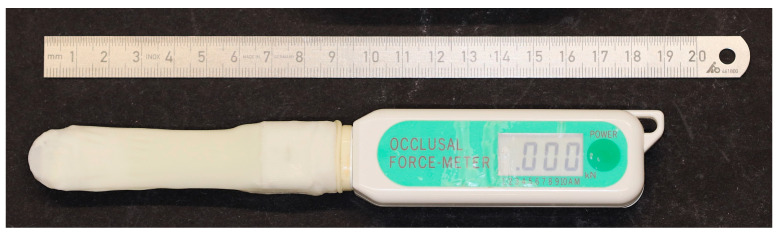
The Occlusal Force Meter GM 10 (OFM) for measuring the bite force in newtons with a hygienic protective cover.

Bite force was measured with the Occlusal Force Meter GM10 (Morita, Nagano Keiki, Higashimagome, Ohta-ku, Tokyo, Japan) (referred to as the OFM) (Figure 1) as an indicator of the functional state of the masticatory muscles. The OFM was used in numerous studies to assess bite force [22,29,30,31]. Measurements following the literature [32] and the manufacturer instructions were performed using the OFM in buccal–oral alignment on the right and left sides of the jaw. The measurements were conducted three times on each side of the jaw, targeting the first molar or the nearest area, with the participants exerting maximum jaw-closing force. If there was no antagonistic contact in this region, the nearest antagonistic contact was used for the measurement. The mean value was calculated from the three measurements per side and used for the analysis. The reason for using three measurements per side was to capture natural variations in the bite force and derive a more representative measure. While participants may indeed be cautious on the first trial, averaging the three values allows for a stable and reproducible measure that better reflects the typical masticatory performance than a single maximum value.

The accuracy of the Occlusal Force Meter (OFM) was previously validated by comparing its measurements with those obtained using a universal testing machine (Retroline Z010, ZwickRoell, Ulm, Germany), which served as a reference standard [33]. In that study, forces ranging from 0 to 700 N were applied to the OFM by the Zwick machine in accordance with the manufacturer’s specifications. The deviations between the applied and recorded values (at a loading speed of 0.3 mm/min) were modeled using polynomial functions. The resulting correction coefficients allowed for the calibration of the OFM to enhance its measurement precision [33].

In the present study, however, the uncorrected OFM readings—hereafter referred to as “theoretical values”—were used for the primary analyses. This decision was made to ensure methodological consistency with previous studies employing the OFM without calibration. Specifically, these values were used in part A to establish reference ranges for bite force according to prosthetic restoration type, and in Part B to evaluate the association between prosthetic restoration and bite force as an objective indicator of masticatory muscle function.

For transparency and future comparability, the calibrated values—termed “effective values”—that incorporate the correction coefficients derived from the Zwick reference are provided in Supplementary Materials I (Table S1 and Figure S1). This enables subsequent studies to compare the findings across both corrected and uncorrected datasets, depending on their methodological alignment.

Additionally, the socio-demographic data of the participants were recorded. All measurements and oral examinations were performed by a single dentist.

To ensure comparability with the established epidemiological data and to enable structured analysis, the prosthetic treatment groups (PTGs) used in this study were defined a priori based on the hierarchical classification system employed in the 4th and 5th German Oral Health Studies (DMS IV and V) [34,35]. All participants were subsequently recruited to match these predefined categories, such that a balanced distribution across the seven prosthetic treatment groups could be achieved.

This classification resulted in seven PTGs, which were further clustered into three main categories according to dental and prosthetic status:

### 2.4. Fully Dentate

PTG 1  Fully dentate, no missing teeth, only natural dentition
PTG 2  Fully dentate, crown or fixed partial denture

### 2.5. Partially Dentate

PTG 3  Partially dentate, no denture
PTG 4  Partially dentate, removable denture
PTG 5  Partially dentate in one jaw, and edentulous with a complete denture in the other jaw

### 2.6. Edentulous

PTG 6  Edentulous, complete dentures in both jaws, supported by two interforaminal implants in the lower jaw
PTG 7  Edentulous, complete dentures in both jaws

### 2.7. Statistical Considerations

Statistical analyses were performed using SPSS (version 27.0, IBM, Chicago, IL, USA) [36]. A descriptive statistical analysis was performed for the absolute frequencies. To compare the different groups, an analysis of variance (ANOVA) was performed. The significance level was set at *p* < 0.05.

In accordance with the STROBE guidelines, missing data were addressed by employing mean imputation. For any missing values within the dataset, the mean of the corresponding variable was calculated and used to replace the missing entry. This method was chosen to minimize the potential bias caused by missing data while maintaining the integrity of the analysis. The imputation process was applied consistently across all relevant variables, and sensitivity analyses were conducted to evaluate the impact of this approach on the study results.

### 2.8. Ethical Consideration

This study was approved by the competent ethics committee of the University of Leipzig (number: 048/21-ek). All participants or their legal representatives gave written informed consent.

## 3. Results

A total of 198 participants (sex: male *n* = 98, 49.5%; female *n* = 100, 50.5%; age: median, 72.0 years (range: 20–95 years)) were included in the study and assigned to the seven PTGs (Table 1). The participants’ dental and prosthetic status characteristics are shown in Table 2.

**Figure 2:**
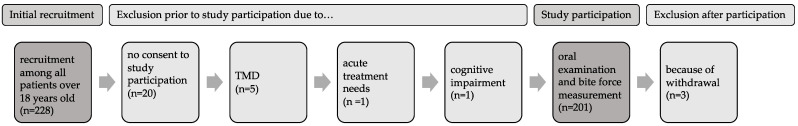
The participant recruitment process, including the number of individuals and the reasons and numbers of drop-outs due to non-participation, exclusion, or withdrawal.

A flow diagram (Figure 2) illustrates the participant recruitment process, including the number of individuals as well as the reasons for non-participation, detailing exclusions and withdrawals.

**Study part A** **—The normative values of the bite force according to the prosthetic leading group.**

The normative values shown in Table 3 were evaluated for the PTGs established in the study.

**Figure 3:**
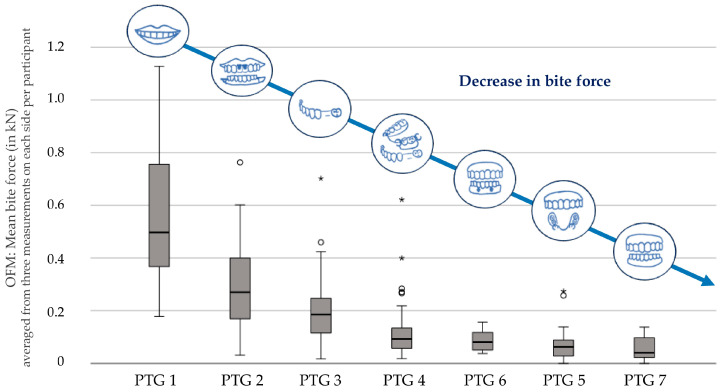
A ranking of the prosthetic treatment groups (PTGs) according to their perceived effects on the bite force. The boxplots display the bite force as the mean of the theoretical occlusal force values, measured for each participant by the Occlusal Force Meter GM10 (OFM) three times each on the right and left sides of the jaw, in kilonewtons (kN). (PTG 1—Fully dentate, no missing teeth, only natural dentition; PTG 2—Fully dentate, crown or fixed partial denture; PTG 3—Partially dentate, no denture; PTG 4—Partially dentate, removable denture; PTG 5—Partially dentate in one jaw, and edentulous with a complete denture in the other jaw; PTG 6—Edentulous, complete dentures in both jaws, supported by two interforaminal implants in the lower jaw; PTG 7—Edentulous, complete dentures in both jaws). (°/*—outliers).

It was shown that the bite force decreased with the decrease in the number of teeth and the change from fixed dentures to removable dentures. However, this did not apply to PTG 6: the participants in this group showed higher bite forces, despite missing teeth, than the participants who were partially edentulous in one jaw and had a full denture in the other jaw (PTG 5). Accordingly, it was possible to rank the PTGs based on their perceived effects on the bite force (Figure 3).

**Study part B** **—The relationship between the prosthetic treatment group and the bite force.**

**Figure 4:**
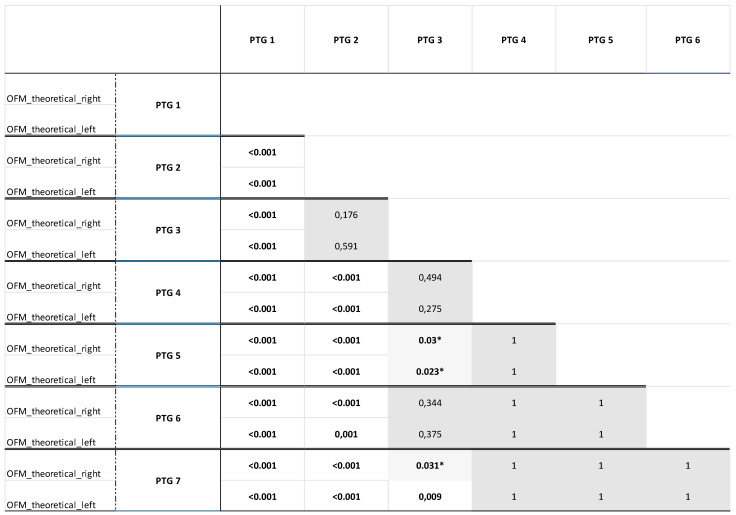
The relationship between the prosthetic treatment group (PTG) and the bite force measured with the Occlusal Force Meter GM10 (OFM) as theoretical measured values (displayed by the OFM = OFM_theoretical) in the different jaw sides (right, left). (PTG 1—Fully dentate, no missing teeth, only natural dentition; PTG 2—Fully dentate, crown or fixed partial denture; PTG 3—Partially dentate, no denture; PTG 4—Partially dentate, removable denture; PTG 5—Partially dentate in one jaw, and edentulous with a complete denture in the other jaw; PTG 6—Edentulous, complete dentures in both jaws, supported by two interforaminal implants in the lower jaw; PTG 7—Edentulous, complete dentures in both jaws). (* indicate that no clear statements can be made in this regard; bold values indicate statistical significant values).

The theoretical measured occlusal force values from the OFM [33] were compared for each PTG with all other PTGs using ANOVA (Figure 4). The results were as follows:

- Full dentition (PTG 1) was considered to be the gold standard regarding the evaluated bite forces, as it was shown that the bite forces of the fully dentate participants were higher than those of all other PTGs, from which they also differed significantly (*p* < 0.001).
- The fully dentate participants with crown(s) or fixed partial denture(s) (PTG 2) did not differ in terms of the measured bite forces from those with partial dentition without dentures (PTG 3) (Figure 4, grey highlighting).
- Similarly, there was no difference in the bite forces between the partially dentate participants without dentures (PTG 3) and the partially dentate participants with removable dentures (PTG 4) (Figure 4, grey highlighting) or the edentulous participants with complete dentures in both jaws supported by two interforaminal implants in the lower jaw (PTG 6) (Figure 4, grey highlighting). Based on the theoretical OFM measurements, no significant differences were observed between the PTGs “partially dentate in one jaw, edentulous with the complete denture in one jaw” (PTG 5) and “edentulous, complete dentures in both jaws” (PTG 7) (Figure 4, grey highlighting). This suggests that similar bite forces can be achieved with partial dentition, whether it is restored or not, as with partial dentition using removable dentures or further reduced dentition requiring greater prosthetic restoration.
- The same applies to all other prosthetic PTGs: there were no differences in the bite forces between the partially dentate participants with removable dentures (PTG 4), the participants partially dentate in one jaw and edentulous with complete dentures in the other jaw (PTG 5), the edentulous participants with complete dentures in both jaws (PTG 7), and the edentulous participants with complete dentures in both jaws, supported by two interforaminal implants in the lower jaw (PTG 6).

The significant values indicate a difference between the PTGs in terms of the bite forces, while the non-significant values (dark grey highlighted) reflect no difference in this respect.

The values with a * indicate that no clear statements can be made in this regard. There was a difference or no difference between the PTGs in relation to the bite force depending on the side of the measurement (light grey highlighted).

## 4. Discussion

For the prosthetic treatment groups defined in the study, the normative values for the bite force (measured with the OFM) were determined. A statistically significant difference was found between the bite forces of the hierarchically established prosthetic treatment groups. Therefore, the null hypothesis (H_0_), which stated that there would be no significant difference in the bite force between the different prosthetic treatment groups, was rejected. This indicates that bite force varies significantly depending on the type of prosthetic restoration and dental status.

### 4.1. Comparison of the Results

Bite force is considered an objective indicator of oral function, and helps to determine masticatory performance. In reconstructive dentistry, the assessment of bite force is therefore considered an important step in diagnosis and treatment planning. However, it does not provide any information regarding the efficiency of the chewing process [37]. Despite the clinical importance of bite force, there is still a lack of comprehensive data comparing different prosthetic rehabilitation strategies in a systematic manner. This study aimed to address this gap by evaluating bite force across a broad spectrum of dental conditions and prosthetic restorations.

To date, no study has assessed bite force across all types of dental conditions and dentures to explore this relationship. Therefore, comparison is limited to specific prosthetic leading groups.

The results of this study show a mean bite force of 547 ± 240 N, which is somewhat lower than the values reported in previous studies, where the mean bite force values ranged from 738 ± 209 N [38] to 619.84 ± 24.17 N [39] in fully dentate subjects. While these results may appear to differ, several factors could explain these variations. These include differences in the sample populations (such as age, sex, and overall dental health), variations in the experimental setups (including the measurement conditions and methodology), and the inclusion of participants with diverse prosthetic treatment statuses in our study. Furthermore, the broader range of bite force values in our study (547 ± 240 N) suggests significant individual variation among the participants. Despite these differences, the consistency of the results within our study and the alignment with other studies using the Occlusal Force Meter (OFM) indicate that this measurement method is reliable and simple.

A correlation between bite force and the number of teeth has been reported in previous studies. Occlusal contact is considered to be the most important factor influencing bite force [19]. Bite force correlates significantly with the Eichner index, the number of remaining teeth, and the tooth condition [40,41,42,43,44]. Gibbs et al. (2002) evaluated a mean bite force of 462 N (range: 98–1031 N) for participants aged 28 to 76 with posterior tooth loss (PTG 3—partially dentate, no dentures) [43], which is comparatively higher than the median masticatory force measured in this study at 203 ± 142 N with the OFM. Some reasons for this may be that a different measuring device was used, and that the measurements were taken in a different region (“clenching forces were supported by first and second molars and second premolars when possible” [43]) than in the present study. In addition, the age group of the participants in Gibbs et al. (2002) [43] is presented in a manner different from the current study, with no information on the median age, etc., presented.

The effect of dentures on the maximum chewing force has been investigated in several studies. The results of this study show that the poorer the dental status, the lower the bite force, which is consistent with a previous study [17]. Al-Zarea et al., 2015 showed that wearing fixed dentures on one side only insignificantly reduced the mean MBF from 596.2 ± 76.3 N to 580.9 ± 74.3 N compared to the side with natural dentition [30]. In this study, the mean bite force values for PTG 1 (fully dentate, no missing teeth, only natural dentition) are similar (547 ± 240 N). However, the bite force values of this study for PTG 2 (fully dentate, crown or fixed partial denture), with 294 ± 164 N, do not correspond to the values from the above-mentioned study by Al-Zarea et al., 2015 [30], although the same measuring instrument (OFM) was used. This discrepancy may be attributed to various sources of bias present in both studies. The potential biases include differences in the sample populations, such as variations in age, sex, or overall health status, as well as potential differences in the study protocols (e.g., how the dentures were placed and the duration of wear). Additionally, the methods of measuring bite force may have been influenced by patient cooperation and other contextual factors, which could contribute to the observed differences in results. These considerations highlight the complexity of studying the effects of prosthetic restorations on bite force and underscore the need for standardized protocols to minimize bias in future studies.

Fixed partial dentures have only 80%, removable partial dentures 35%, and complete dentures 11% of the maximum bite force of participants with natural teeth [45]. Further studies confirm the decrease in bite force in this order [46,47]. Values of 464.24 ± 17.15 N for natural dentition, 297.15 ± 28.85 N for partial dentures, and 280.42 ± 47.71 N for complete dentures have been reported in the literature [47]. Since the measurements were carried out with the Prescale II (Dental Prescale II, GC Corporation, Tokyo, Japan), a comparison to our study is not possible.

Several studies have shown that people with complete dentures have the lowest chewing force of all PTGs (Fayad et al., 2018: 34 N [48]; Alqutaibi et al., 2023: 123.5 N [49]; Sônego et al., 2022: 5.4 kgf (≙53 N) [50]; and Kurogi et al., 2023: MBF 53.5N (range 25.3–81.7) [51]). These bite force values are consistent with those reported in this study.

While the MBF in untreated edentulism is 5.1 ± 2.6 N, prosthetic treatment with a complete denture can improve the value to 44.8 ± 15.2 N [52]. Treatment with an implant-supported overdenture for edentulism improved the MBF compared to a complete denture (Rismanchian et al., 2009: 122.2 N to 370.4 N [53]; Soni et al., 2020: 81 N to 214 N [54]; Sharma et al., 2017: 62.9 N (31—88 N) to 132.2 N (57—192 N) [55]). When comparing the bite forces (MBF) of different prosthetic configurations, significant differences were observed. For example, when a complete denture was worn in the maxilla and an overdenture in the mandible, MBF values of 119.8 ± 265 N were recorded, compared to 68.7 ± 20.6 N for complete dentures in both jaws [53]. Studies have shown that implant-supported dentures can significantly increase MBF. Possebon et al. [56] reported that the MBF for implant-supported dentures was 127.52% higher (72.9 ± 51.0 N) compared to full dentures (32.1 ± 8.8 N), which was not confirmed in our study.

### 4.2. Study Limitations

The study’s limitations primarily stem from the following factors: In the analysis and prioritization of the bite force, only the dependence on PTGs was considered. Other factors, such as cognition [57], the presence of sarcopenia [58], age [59], and gender [60], were not taken into account. These variables could potentially influence the measured bite forces, resulting in a reduction in bite force. Given the lack of a clear age structure for the prosthetic guide groups in this study, the authors assume that the impact of age (except for PTG 1) is minimal. The gender distribution was nearly equal, except for PTGs 3 and 6. Cognitive deficits were not assessed. It is presumed that with increasing age, their prevalence rises [61]. As PTGs 2—6 showed no significant differences in age distribution, this factor is also considered a subordinate influencing factor. PTG 1 exhibited the greatest variance in bite force values, with the participants being the youngest on average.

In addition to the specified prosthetic leading groups, other scenarios, such as implant-supported prostheses in a fully edentulous arch, were not investigated. This decision was made because including additional groups, such as fully edentulous patients with implant-supported prostheses, would have required a larger sample size and a broader study design. The focus of this study was on comparing the bite forces across the specified prosthetic groups, including partially edentulous patients with two implants in the mandible. This needs clarification in subsequent research. An increase in bite force and a decrease in interocclusal tactility are expected with fixed implant-supported dentures. In implant-supported fixed restorations, a decrease in interocclusal tactility is assumed. The underlying process of osseoperception leads to an unfavorable modulation of the masticatory process. Consequently, the applied jaw-closing forces increase with this type of restoration. The reduced or absent feedback and resulting high jaw-closing forces present challenges regarding the potential mechanical complications (increased risk of prosthetic restoration fractures). Patients often arbitrarily or unconsciously reduce their jaw-closing force to prevent fractures of the restoration. This can result in reduced chewing efficiency [62,63].

Previous studies [17,38,39,45,46,47,48,49,50,51,53,54,55] have focused on individual PTGs, making comparisons with the literature difficult and precluding the calculation of statistical power for the current study.

Another limitation to consider is the bite force measurement device used. In a prior study by the authors, it was found that measuring absolute values with this device is challenging, and tracking values over time appears more ideal [33]. Commercially available instruments for measuring bite force, such as pressure-sensitive foils like the T-Scan-System and Dental Prescale, as well as strain gauges or pneumatic pressure transducers like MPX5700 [64], were not suitable for the conducted measurements (e.g., no display of absolute values). The device employed in this study is the GM10 Occlusal Force-Meter (NAGANO KEIKI Co., Ltd., Tokyo, Japan), comprising a vinyl bite plate with a hydraulic pressure gauge. It is highly suitable for clinical use due to its size, weight, and easy handling.

In the literature, correlations between chewing laterality and lateral differences in bite force have been observed in previous studies [39,65,66]. The studies show an increase in the bite force from the incisors to the premolars, and then to the molars [67,68]. Since the OFM measurements are required to be taken in the region of the first molar, it can be assumed that the recorded values represent the maximum bite force for each participant.

It is noteworthy that a single examiner conducted all clinical examinations and measurements in this study, ensuring a degree of consistency. However, due to the study design, blinding of both the examiner and the participants was not feasible.

Future studies should also incorporate a longitudinal approach to assess how prosthetic rehabilitation affects bite force over time, and whether patients adapt functionally to their prosthetic restorations. Moreover, the integration of electromyography (EMG) could provide deeper insights into neuromuscular adaptations in response to different prosthetic treatments.

### 4.3. Important Results of This Study

The results indicate that participants with full dentition exhibit significantly higher bite forces compared to all other prosthetic treatment groups (PTGs), reinforcing the notion that a complete set of natural teeth represents the gold standard for occlusal force. However, the findings also reveal no significant differences in the bite forces between various groups with partial dentition, regardless of whether they received prosthetic treatment. Specifically, there were no differences in the bite forces between the participants with partial dentition without any prosthetic restoration and those with fixed or removable dentures.

These findings challenge the assumption that prosthetic rehabilitation always leads to a functional improvement in terms of the bite force. While prosthetic treatment remains crucial for aesthetics, phonetics, and comfort, its direct impact on bite force appears to be more complex and multifactorial than previously assumed.

This suggests that although a decrease in the number of natural teeth and an increase in the degree of prosthetic restoration generally lead to a reduction in bite force, a supposedly better prosthetic treatment (e.g., partial dentition with removable dentures compared to untreated partial dentition) does not necessarily result in an improved bite force. Therefore, when considering the bite force alone, the differences between the PTGs are not substantial enough to justify prosthetic intervention in every case. However, it is important to note that other factors that could influence the choice of treatment were not investigated in this study.

Future research should focus on patient-reported outcomes and chewing efficiency rather than bite force alone to gain a more comprehensive understanding of the functional benefits provided by different prosthetic solutions.

## 5. Conclusions

In conclusion, the study results indicate that oral status significantly influences bite force, and thus chewing function. The measurement of bite force, as used in this study, proves to be a reliable and objective parameter for evaluating chewing function in patients with various prosthetic restorations. The results suggest that prosthetic rehabilitation can have a measurable impact on bite force, with variations observed depending on the type of restoration used. However, the relationship between bite force and malnutrition, particularly in the context of sarcopenia, requires further investigation and was not directly addressed in this study.

While the study does not directly link bite force to nutritional status, it highlights the importance of assessing the chewing function as part of comprehensive dental care, particularly in elderly patients. Future research could explore how changes in bite force correlate with nutritional outcomes in patients with different prosthetic restorations.

## Figures and Tables

**Table 1:** The participants’ socio-demographic characteristics in total and by the seven prosthetic treatment groups (PTGs).

	All Participants	PTG 1	PTG 2	PTG 3	PTG 4	PTG 5	PTG 6	PTG 7
		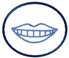	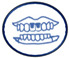	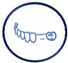	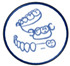	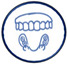	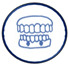	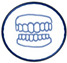
		Fully dentate, no missing teeth, only natural dentition	Fully dentate, crown or fixed partial denture	Partially dentate, no denture	Partially dentate, removable denture	Partially dentate in one jaw, and edentulous with a complete denture in the other jaw	Edentulous, complete dentures in both jaws, supported by two interforaminal implants in the lower jaw	Edentulous, complete dentures in both jaws
	(*n* = 198)	(*n* = 21)	(*n* = 40)	(*n* = 37)	(*n* = 46)	(*n* = 25)	(*n* = 12)	(*n* = 17)
Sex (*n*%)								
Male	98/49.5	9/42.9	17/42.5	25/67.6	21/45.7	12/48.0	4/33.3	10/58.8
Female	100/50.5	12/57.1	23/57.5	12/32.4	25/54.3	13/52.0	8/66.7	7/41.2
Age (Years)								
Median (Range)	72.0 (20–95)	22 (20–33)	69.5 (24–89)	71 (33–93)	75 (46–90)	77 (34–94)	81.5 (69–95)	79 (47–94)
Mean ± SD	66.1 ± 20.6	23.2 ± 3.7	64.5 ± 20.1	70.2 ± 14.2	72.7 ± 10.5	73.5 ±15.1	81.0 ± 8.5	74.1 ± 12.8

**Table 2:** The participants’ dental and prosthetic characteristics in total and by the seven prosthetic treatment groups. (D—decayed, M—missing, F—filled, T—teeth; * Assessment according to Markorxs, only possible for denture wearers).

	All Participants	PTG 1	PTG 2	PTG 3	PTG 4	PTG 5	PTG 6	PTG 7
		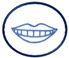	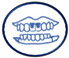	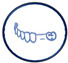	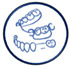	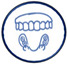	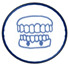	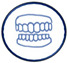
		Fully dentate, no missing teeth, only natural dentition	Fully dentate, crown or fixed partial denture	Partially dentate, no denture	Partially dentate, removable denture	Partially dentate in one jaw, and edentulous with a complete denture in the other jaw	Edentulous, complete dentures in both jaws, supported by two interforaminal implants in the lower jaw	Edentulous, complete dentures in both jaws
**DMFT index**								
DT								
Median (Range)	0 (0–19)	0 (0–1)	0 (0–19)	1 (0–15)	0 (0–12)	0 (0–6)	0 (0–0)	0 (0–0)
Mean ± SD	1.2 ± 2.7	0.1 ± 0.3	1.0 ± 3.2	2.6 ± 3.6	1.7 ± 2.8	0.7 ± 1.6	0 ± 0	0 ± 0
MT								
Median (Range)	13 (0–32)	2 (0–6)	5 (0–17)	8 (1–31)	15.5 (2–27)	28 (18–31)	32 (30–32)	32 (32–32)
Mean ± SD	14.9 ± 10.7	2.1 ± 2.0	6.1 ± 3.3	9.5 ± 6.1	15.8 ± 4.7	26.6 ± 3.8	32.0 ± 0	31.9 ± 0.5
FT								
Median (Range)	8 (0–26)	1 (0–5)	13.5 (3–23)	16 (0–26)	11 (0–22)	1 (0–13)	0 (0–0)	0 (0–0)
Mean ± SD	8.4 ± 7.2	1.3 ± 1.5	13.0 ± 5.2	14.0 ± 6.8	10.9 ± 5.0	3.4 ± 3.6	0 ± 0	0 ± 0
DMFT								
Median (Range)	28 (0–32)	4 (0–11)	20.5 (6–30)	28 (16–32)	29 (19–32)	32 (23–32)	32 (30–32)	32 (30–32)
Mean ± SD	24.5 ± 9.2	3.6 ± 2.7	20.2 ± 6.2	26.1 ± 5.2	28.4 ± 3.5	30.6 ± 2.3	32.0 ± 0	31.9 ± 0.5
**Number of support zones**								
Median (Range)	4 (0–4)							
Mean ± SD	3.7 ± 0.8							
(*n*/%)								
None	4/2.0			3/8.1	1/2.2			
One	2/1.0			1/2.7		1/4.0		
Two	12/6.1			7/18.9	3/6.5	2/8.0		
Three	10/5.1		1/2.5	7/18.9	1/2.2	1/4.0		
Four	170/85.9	21/100	39/97.5	19/51.4	41/89.1	21/84.0	12/100	17/100
**Eichner index** (*n*/%) (*n* = 198)								
A1	53/26.6	21/100						
A2	16/8.0			11/29.7	1/2.2			
A3	9/4.5			6/16.2	1/2.2			
B1	13/6.5		32/80.0	9/24.3	2/4.3	1/4.0		
B2	24/12.1		4/10.0	7/18.9	16/34.8			
B3	11/5.5		2/5.0	1/2.7	10/21.7			
B4	9/4.5		1/2.5	1/2.7	8/17.4			
C1	11/5.5		1/2.5		8/17.4	2/8.0		1/8.3
C2	34/17.1			2/5.4		22/88.0		10/83.3
C3	18/9.0						17/100	1/8.3
**Denture quality according to Marxkors** (*n*/%) (*n* = 100)								
1	47/47.0	*	*	*	27/58.7	9/36.0	5/29.4	6/50.0
2	27/27.0				11/23.9	9/36.0	2/11.8	5/41.7
3	18/18.0				6/13.0	4/16.0	7/41.2	1/8.3
4	8/8.0				2/4.3	3/12.0	3/17.6	

**Table 3:** The reference values of the bite force in newtons (N), for all participants and by prosthetic treatment group (PTG), measured with the Occlusal Force Meter GM10 (OFM) on the right and left sides of the jaw. (SD—standard deviation) (* different *n* compared to the total number of participants within the prosthetic treatment group, resulting from the ability or inability of the participants to bite on the corresponding side due to their dental and denture status).

	All Participants	PTG 1	PTG 2	PTG 3	PTG 4	PTG 5	PTG 6	PTG 7
		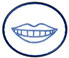	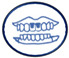	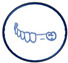	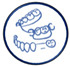	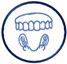	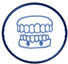	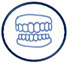
		Fully dentate, no missing teeth, only natural dentition	Fully dentate, crown or fixed partial denture	Partially dentate, no denture	Partially dentate, removable denture	Partially dentate in one jaw, and edentulous with a complete denture in the other jaw	Edentulous, complete dentures in both jaws, supported by two interforaminal implants in the lower jaw	Edentulous, complete dentures in both jaws
**OFM right** (in N)	*n* = 193			*n* = 33 *	*n* = 45 *			
Median (Range)	142 (0–1163)	490 (109–1163)	257 (19–901)	158 (17–779)	104 (14–657)	59 (0–288)	75 (32–152)	38 (0–184)
Mean ± SD	214 ± 218	574 ± 260	310 ± 187	212 ± 180	129 ± 121	77 ± 74	83 ± 38	60 ± 56
**OFM left** (in N)	*n* = 190			*n* = 31 *	*n* = 45 *	*n* = 24 *		
Median (Range)	124 (0–1093)	440 (197–1093)	255 (27–666)	171 (26–625)	81 (15–586)	68 (0–259)	87 (26–162)	44 (0–131)
Mean ± SD	197 ± 195	520 ± 236	277 ± 164	203 ± 149	121 ± 119	77 ± 66	89 ± 44	50 ± 43
**Mean OFM right/left** (in N)	*n* = 194							
Mean ± SD	204 ± 199	547 ± 240	294 ± 164	203 ± 142	125 ± 111	75 ± 69	86 ± 40	55 ± 45

## Data Availability

The data presented in this study are available on request from the corresponding author. The data are not publicly available due to privacy.

## Supplementary Materials

The following supporting information can be downloaded at https://www.mdpi.com/article/10.3390/jcm14082723/s1; Table S1: The reference values of the bite force (maximal occlusal force), measured with the Occlusal Force Meter GM10 (OFM) as effective occlusal forces of the OFM determined using the ZWICK machine and calculated using polynomial coefficients [33], on the right and left sides of the jaw in newtons (N) for all participants and by prosthetic treatment group (PTG) (SD—standard deviation); Figure S1: The relationship between the prosthetic treatment group (PTG) and the bite force measured with the Occlusal Force Meter GM10 (OFM) on different jaw sides (right, left) as effective measured values (OFM_effective), determined using the ZWICK machine and calculated using polynomial coefficients.

## Abbreviations

The following abbreviations are used in this manuscript:

OFM	Occlusal Force Meter
PTG	Prosthetic treatment group
