# Supplementary material for: An Evaluation of Reference Bite Force Values: Investigating the Relationship Between Dental Prosthetic Restoration and Bite Force in a Cross-Sectional Study
Source: J Clin Med. 2025 Apr 15;14(8):2723. doi: 10.3390/jcm14082723 (PMC12027650; doi:10.3390/jcm14082723)
Supplement: Supplementary file 1 [file jcm-14-02723-s001.zip › jcm-3551496-supplementary.pdf]

Supplementary Information

Table S1. Reference values of bite force (maximal occlusal force) measured with the Occlusal Force Meter GM10 (OFM) as effective occlusal forces of the OFM determined using the ZWICK machine and calculated using polynomial coefficients [33] on the right or left side of the jaw in newton (N) for all participants and by prosthetic treatment groups (PTG) (SD – standard deviation) (*different n compared to the total number of participants within the prosthetic treatment group result from the ability or inability of the participants to bite on the corresponding side due to their dental and denture status)

	**All participants**	**PTG 1**	**PTG 2**	**PTG 3**	**PTG 4**	**PTG 5**	**PTG 6**	**PTG 7**
		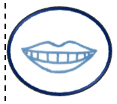	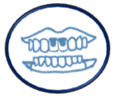	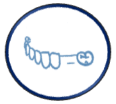	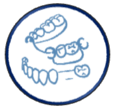	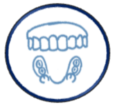	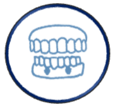	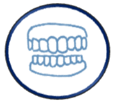
		**Fully dentate, no missing teeth, only natural dentition**	**Fully dentate, crown or fixed partial denture**	**Partially dentate, no denture**	**Partially dentate, removable dentur**	**partially dentate in one jaw, and edentulous with complete denture in the other jaw**	**Edentulous, complete dentures in both jaws, supported by two interforaminal implants in lower jaw**	**Edentulous, complete dentures in both jaws**
	(n=198)	(n=21)	(n=40)	(n=37)	(n=46)	(n=25)	(n=12)	(n=17)
**OFM effective right (in N)**								
**Median (Range)**	192 (0-1916)	622 (180-1916)	365 (48-1488)	221 (18-1289)	134 (14-1090)	85 (0-487)	97 (46-192)	73 (0-267)
**Mean ± SD**	298 ± 311	761 ± 1736	435 ± 281	292 ± 269	198 ± 205	112 ±122	108 ± 46	84 ± 271
**OFM effective left (in N)**								
**Median (Range)**	164 (0-1802)	564 (282-1802)	357 (74-1038)	234 (29-1038)	118 (23-974)	91 (0-440)	112 (38-204)	67 (0-161)
**Mean ± SD**	276 ± 277	693 ± 1520	392 ± 242	273 ± 211	188 ± 202	112 ± 110	115 ± 54	72 ± 53
**Mean OFM effective right/left (in N)**								
**Mean ± SD**	285 ± 284	727 ± 368	414 ± 249	276 ± 215	193 ± 190	110 ± 115	111 ± 49	78 ± 59

		PTG 1<br>Fully dentate, no missing teeth, only natural dentition	PTG 2<br>Fully dentate, crown or fixed partial denture	PTG 3<br>Partially dentate, no denture	PTG 4<br>Partially dentate, removable denture	PTG 5<br>Partially dentate in one jaw, edentulous with complete denture in one jaw	PTG 6<br>Edentulous, complete dentures in both jaws, supported by two interforaminal implants in lower jaw	PTG 7<br>Edentulous, complete dentures in both jaws
OFM_effective_right	**PTG 1<br>Fully dentate, no missing teeth, only natural dentition**							
OFM_effective_left								
OFM_effective_right	**PTG 2 Fully dentate, crown or fixed partial denture**	**<0.001**						
OFM_effective_left		**<0.001**						
OFM_effective_right	**PTG 3 Partially dentate, no denture**	**<0.001**	0,262					
OFM_effective_left		**<0.001**	0,431					
OFM_effective_right	**PTG 4 Partially dentate, removable denture**	**<0.001**	**<0.001**	1				
OFM_effective_left		**<0.001**	**<0.001**	1				
OFM_effective_right	**PTG 5 Partially dentate in one jaw, edentulous with complete denture in one jaw**	**<0.001**	**<0.001**	0.118*	1			
OFM_effective_left		**<0.001**	**<0.001**	0.121*	1			
OFM_effective_right	**PTG 6 Edentulous, complete dentures in both jaws, supported by two interforaminal implants in lower jaw**	**<0.001**	**0,001**	0,525	1	1		
OFM_effective_left		**<0.001**	**0,002**	0,618	1	1		
OFM_effective_right	**PTG 7<br>Edentulous, complete dentures in both jaws**	**<0.001**	**<0.001**	0.092*	1	1	1	
OFM_effective_left		**<0.001**	**<0.001**	**0,042**	1	1	1	

Figure S1. Relationship between the prosthetic treatment group (PTG) and the bite force measured with the Occlusal Force Meter GM10 (OFM) in different jaw sides (right, left) as effective measured values (OFM_effective) determined using the ZWICK machine and calculated using polynomial coefficients [33].

Significant values indicate a difference between the PTG in terms of bite forces, while non-significant values (dark grey highlighted) reflect no difference in this respect.

Values with a * indicate that no clear statements can be made in this regard. There was a difference or no difference between the PTG in relation to the bite force depending on the side of the measurement (light grey highlighted).
